# Crystal structure of the co-crystalline adduct 1,3,6,8-tetra­aza­tri­cyclo­[4.4.1.1^3,8^]dodecane (TATD)–4-bromo­phenol (1/2)

**DOI:** 10.1107/S2056989015006684

**Published:** 2015-04-09

**Authors:** Augusto Rivera, Juan Manuel Uribe, Jicli José Rojas, Jaime Ríos-Motta, Michael Bolte

**Affiliations:** a Universidad Nacional de Colombia, Sede Bogotá, Facultad de Ciencias, Departamento de Química, Cra 30 No. 45-03, Bogotá, Código Postal 111321, Colombia; b Institut für Anorganische Chemie, J. W. Goethe-Universität Frankfurt, Max-von Laue-Strasse 7, 60438 Frankfurt/Main, Germany

**Keywords:** crystal structure, co-crystalline adducts, TATD, proton transfer, hydrogen bonding

## Abstract

The structure of the 1:2 co-crystalline adduct C_8_H_16_N_4_·2C_6_H_5_BrO, (I), from the solid-state reaction of 1,3,6,8-tetra­aza­tri­cyclo­[4.4.1.1^3,8^]dodecane (TATD) and 4-bromo­phenol, has been determined. The asymmetric unit of the title co-crystalline adduct comprises a half mol­ecule of aminal cage polyamine plus a 4-bromo­phenol mol­ecule. A twofold rotation axis generates the other half of the adduct. The primary inter-species association in the title compound is through two inter­molecular O—H⋯N hydrogen bonds. In the crystal, the adducts are linked by weak non-conventional C—H⋯O and C—H⋯Br hydrogen bonds, giving a two-dimensional supra­molecular structure parallel to the *bc* plane.

## Abstract 2

The first crystal structure determination of a 1:2 co-crystalline adduct of 1,3,6,8-tetra­aza­tri­cyclo­[4.4.1.1^3,8^]dodecane is presented. In the crystal, adducts are linked by C—H⋯O and C—H⋯Br hydrogen bonds, forming a two-dimensional network.

## Chemical context   

The main focus of the research in our laboratory is the synthesis of a variety of mol­ecules using cyclic aminals of the adamantane type. The prototype of these reactions is a Mannich-type reaction involving 1,3,6,8-tetra­aza­tri­cyclo­[4.4.1.1^3,8^] dodecane (TATD) (II) with phenols which, in solution, affords di-Mannich bases of type (III) (Rivera *et al.*, 1993 ▸, 2005 ▸). These are common systems for the investigation of hydrogen bonding and proton transfer. Engaged in the development of greener synthetic pathways, we attempted a synthesis of a di-Mannich base under solvent-free conditions by simply grinding TATD and 4-bromo­phenol at room temperature without using any solvent in the initial step. We found that the reaction did not provide the di-Mannich base as desired. Instead, the title compound, (I), was obtained in good yield. The reaction is run in the absence of solvent, there are no by-products, and the work-up procedure is easy. Recrystallization in an appropriate solvent gave the title compound in high yield.
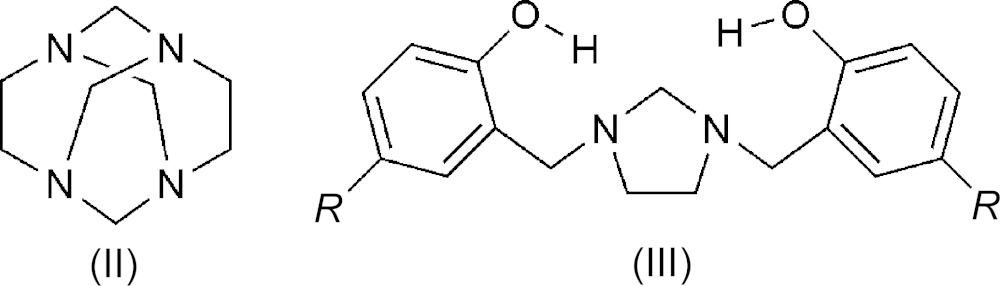


## Structural commentary   

**Figure 1:**
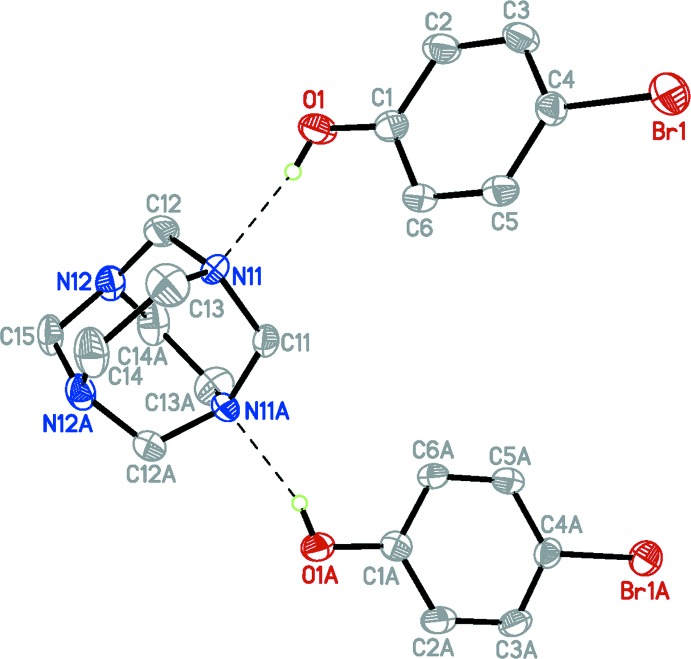
The mol­ecular structure of the title adduct. Displacement ellipsoids are drawn at the 50% probability level. H atoms bonded to C atoms are omitted for clarity. Hydrogen bonds are drawn as dashed lines. Atoms labelled with the suffix A are generated using the symmetry operator (−*x* − 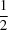, −*y* − 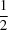, *z*).

Co-crystal (I) crystallized in the space group *Fdd*2 with one half-mol­ecule of 1,3,6,8-tetra­aza­tri­cyclo­[4.4.1.1^3,8^]dodecane (TATD) and one mol­ecule of 4-bromo­phenol in the asymmetric unit; a twofold rotation axis generates the other half of the adduct held together by two inter­molecular O—H⋯N hydrogen bonds [O⋯N 2.705 (5) Å; O—H⋯N 158 (7)°)] (Fig. 1 ▸). Unlike the situation in a related structure (Rivera *et al.*, (2007 ▸), where a 1:1 adduct formed *via* an O—H⋯N hydrogen bond between TATD and hydro­quinone, the title compound features an 1:2 adduct. Bond lengths in the TATD and 4-bromo­phenol mol­ecules in (I) are within normal ranges (Allen *et al.*, 1987 ▸) and are comparable to those found in similar structures (Rivera *et al.*, 2007 ▸; Tse *et al.*, 1977 ▸). The H atom of the phenolic –OH group deviates slightly from the benzene ring plane, subtending a torsion angle of 8(5)°.

A significant reduction in the O⋯N distance is observed when the distance and angle in the O1—H1⋯N1 hydrogen bond [O⋯N 2.705 (5) Å; O—H⋯N 158 (7)°)] in the title compound are compared to the values found in the TATD:hydro­quinone, 1:1 adduct [O⋯N 2.767 (2) Å; O—H⋯N 156.3 (10)°)] (Rivera *et al.*, 2007 ▸). Also, the C1—O1 bond length observed here [1.355 (6) Å], is shorter than that in the hydro­quinone co-crystal. This indicates an increase in hydrogen-bonding strength in the title compound, which may be due to the considerable differences in the p*K_a_* values between the species involved in the hydrogen bond (Majerz *et al.*, 1997 ▸). Compared to hydro­quinone (p*K_a_* = 9.85), *p*-bromo­phenol is more acidic (p*K_a_* = 9.37) (Lide, 2003 ▸).

## Supra­molecular features   

**Figure 2:**
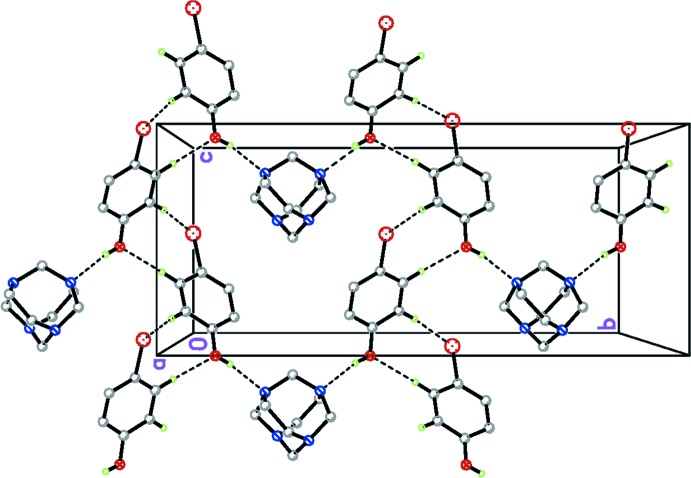
The crystal packing of the title compound, showing two of the chains that extend along the crystal *c*-axis direction. C—H⋯O and C—H⋯Br hydrogen bonds are drawn as dashed lines.

In the crystal of the title compound, the adducts are weakly linked peripherally through both non-conventional C—H⋯O and C—H⋯Br hydrogen bonds (Table 1 ▸) giving a two dimensional supra­molecular structure parallel to the *bc* plane. (Fig. 2 ▸). This is similar to the structure of the 4-bromo­phenol adduct with urotropine (Tse *et al.*, 1977 ▸).

## Database survey   

A database search (CSD version 5.36, November 2014 plus two updates) for 4-bromo­phenol yielded 17 hits with 21 fragments. The mean C—O bond length in these structures is 1.35 (5) Å and the mean C—Br bond length is 1.91 (3) Å. These values are in excellent agreement with those of the title compound, *i.e.* O1—C1 1.355 (6) and Br1—C4 1.907 (5) Å.

A database search for 1,3,6,8-tetra­aza­tri­cyclo­[4.4.1.1^3,8^]dodecane yielded only three hits, two determinations of the compound itself (Murray-Rust, 1974 ▸; Rivera *et al.*, 2014 ▸) and a co-crystal of the aminal with hydro­quinone (Rivera *et al.*, 2007 ▸). While the mol­ecules of 1,3,6,8-tetra­aza­tri­cyclo[4.4.1.1^3,8^]dodecane itself have 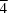
2*m* symmetry, the mol­ecules in the co-crystal of TATD with hydro­quinone have mirror symmetry. In the title compound, on the other hand, the 1,3,6,8-tetra­aza­tri­cyclo­[4.4.1.1^3,8^]dodecane mol­ecule displays *C*_2_ symmetry.

## Synthesis and crystallization   

1,3,6,8-tetra­aza­tri­cyclo­[4.4.1.1^3,8^]dodecane (TATD) (0.21g, 1.25 mmol) and 4-bromo­phenol (0.43g, 2.5 mmol) were manually mixed in a mortar with pestle at room temperature for 20 min as required to complete the reaction (TLC). The mixture was then dissolved in a minimum amount of methanol and left to crystallize at room temperature. Subsequent recrystallization with MeOH then yielded the title compound as white crystals in 78% yield, m.p. = 367–368 K.

## Refinement details   

Crystal data, data collection and structure refinement details are summarized in Table 2 ▸. All the H atoms were located in a difference electron density map. The hydroxyl H atom was refined freely, while C-bound H atoms were fixed geometric­ally (C—H = 0.95 or 0.99 Å) and refined using a riding-model approximation, with *U*_iso_(H) set to 1.2*U*_eq_ of the parent atom.

## Supplementary Material

Crystal structure: contains datablock(s) I. DOI: 10.1107/S2056989015006684/sj5449sup1.cif

Structure factors: contains datablock(s) I. DOI: 10.1107/S2056989015006684/sj5449Isup2.hkl

Supporting information file. DOI: 10.1107/S2056989015006684/sj5449Isup3.cml

CCDC reference: 1057775

Additional supporting information:  crystallographic information; 3D view; checkCIF report

## Figures and Tables

**Table 1:** Hydrogen-bond geometry (, )

*D*H*A*	*D*H	H*A*	*D* *A*	*D*H*A*
O1H1N11	0.78(7)	1.97(7)	2.705(5)	158(7)
C3H3O1^i^	0.95	2.42	3.347(6)	164
C13H13*A*Br1^ii^	0.99	2.89	3.833(6)	159

Symmetry codes: (i) 
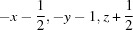
; (ii) 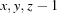.

**Table 2:** Experimental details

Crystal data	
Chemical formula	C_8_H_16_N_4_2C_6_H_5_BrO
*M*_r_	514.27
Crystal system, space group	Orthorhombic, *F* *d* *d*2
Temperature (K)	173
*a*, *b*, *c* ()	20.693(2), 21.7954(18), 9.4649(9)
*V* (^3^)	4268.8(7)
*Z*	8
Radiation type	Mo *K*
(mm^1^)	3.82
Crystal size (mm)	0.29 0.27 0.23

Data collection	
Diffractometer	Stoe *IPDS* II two-circle
Absorption correction	Multi-scan (*MULABS*; Spek, 2009 ▸; Blessing, 1995 ▸)
*T*_min_, *T*_max_	0.847, 0.972
No. of measured, independent and observed [*I* > 2(*I*)] reflections	5997, 1996, 1833
*R*_int_	0.062
(sin /)_max_ (^1^)	0.608

Refinement	
*R*[*F* ^2^ > 2(*F* ^2^)], *wR*(*F* ^2^), *S*	0.032, 0.069, 1.01
No. of reflections	1996
No. of parameters	132
No. of restraints	1
H-atom treatment	H atoms treated by a mixture of independent and constrained refinement
_max_, _min_ (e ^3^)	0.24, 0.41
Absolute structure	Flack *x* determined using 792 quotients [(*I* ^+^)(*I* )]/[(*I* ^+^)+(*I* )] (Parsons *et al.*, 2013 ▸)
Absolute structure parameter	0.003(16)

Computer programs: *X-AREA* (Stoe Cie, 2001 ▸), *SHELXS97* and *XP* in *SHELXTL-Plus* (Sheldrick, 2008 ▸), *SHELXL2014* (Sheldrick, 2015 ▸) and *Mercury* (Macrae *et al.*, 2006 ▸).

## supplementary crystallographic information

### Crystal data

**Table:**

C_8_H_16_N_4_·2C_6_H_5_BrO	*D*_x_ = 1.600 Mg m^−^^3^
*M**_r_* = 514.27	Mo *K*α radiation, λ = 0.71073 Å
Orthorhombic, *F**d**d*2	Cell parameters from 7202 reflections
*a* = 20.693 (2) Å	θ = 3.7–26.0°
*b* = 21.7954 (18) Å	µ = 3.82 mm^−^^1^
*c* = 9.4649 (9) Å	*T* = 173 K
*V* = 4268.8 (7) Å^3^	Block, colourless
*Z* = 8	0.29 × 0.27 × 0.23 mm
*F*(000) = 2080	

### Data collection

**Table:**

Stoe IPDS II two-circle diffractometer	1833 reflections with *I* > 2σ(*I*)
Radiation source: Genix 3D IµS microfocus X-ray source	*R*_int_ = 0.062
ω scans	θ_max_ = 25.6°, θ_min_ = 3.7°
Absorption correction: multi-scan (*MULABS*; Spek, 2009; Blessing, 1995)	*h* = −22→24
*T*_min_ = 0.847, *T*_max_ = 0.972	*k* = −25→26
5997 measured reflections	*l* = −11→11
1996 independent reflections	

### Refinement

**Table:**

Refinement on *F*^2^	Hydrogen site location: mixed
Least-squares matrix: full	H atoms treated by a mixture of independent and constrained refinement
*R*[*F*^2^ > 2σ(*F*^2^)] = 0.032	*w* = 1/[σ^2^(*F*_o_^2^) + (0.0386*P*)^2^] where *P* = (*F*_o_^2^ + 2*F*_c_^2^)/3
*wR*(*F*^2^) = 0.069	(Δ/σ)_max_ < 0.001
*S* = 1.00	Δρ_max_ = 0.24 e Å^−^^3^
1996 reflections	Δρ_min_ = −0.41 e Å^−^^3^
132 parameters	Absolute structure: Flack *x* determined using 792 quotients [(*I*^+^)-(*I*^-^)]/[(*I*^+^)+(*I*^-^)] (Parsons *et al.*, 2013)
1 restraint	Absolute structure parameter: 0.003 (16)

### Special details

**Table:**

Geometry. All e.s.d.'s (except the e.s.d. in the dihedral angle between two l.s. planes) are estimated using the full covariance matrix. The cell e.s.d.'s are taken into account individually in the estimation of e.s.d.'s in distances, angles and torsion angles; correlations between e.s.d.'s in cell parameters are only used when they are defined by crystal symmetry. An approximate (isotropic) treatment of cell e.s.d.'s is used for estimating e.s.d.'s involving l.s. planes.

### Fractional atomic coordinates and isotropic or equivalent isotropic displacement parameters (Å^2^)

**Table:**

	*x*	*y*	*z*	*U*_iso_*/*U*_eq_	Occ. (<1)
Br1	−0.11272 (3)	−0.43922 (2)	1.02771 (5)	0.03734 (18)	
O1	−0.2465 (2)	−0.40437 (17)	0.4684 (4)	0.0311 (9)	
H1	−0.233 (4)	−0.375 (3)	0.432 (7)	0.036 (18)*	
C1	−0.2172 (3)	−0.4091 (2)	0.5959 (5)	0.0232 (11)	
C2	−0.2281 (3)	−0.4622 (2)	0.6734 (6)	0.0288 (12)	
H2	−0.2567	−0.4926	0.6379	0.035*	
C3	−0.1978 (3)	−0.4710 (2)	0.8016 (5)	0.0281 (11)	
H3	−0.2048	−0.5077	0.8536	0.034*	
C4	−0.1570 (3)	−0.4262 (2)	0.8536 (5)	0.0246 (10)	
C5	−0.1468 (2)	−0.37234 (18)	0.7795 (7)	0.0252 (10)	
H5	−0.1190	−0.3416	0.8165	0.030*	
C6	−0.1773 (3)	−0.3635 (2)	0.6514 (5)	0.0257 (11)	
H6	−0.1711	−0.3263	0.6009	0.031*	
N11	−0.2299 (2)	−0.30437 (16)	0.3032 (4)	0.0241 (9)	
N12	−0.2997 (2)	−0.2824 (2)	0.0903 (4)	0.0276 (10)	
C11	−0.2500	−0.2500	0.3809 (7)	0.0278 (16)	
H11A	−0.2137	−0.2379	0.4431	0.033*	0.5
H11B	−0.2863	−0.2621	0.4431	0.033*	0.5
C12	−0.2756 (3)	−0.3255 (2)	0.1929 (6)	0.0354 (14)	
H12A	−0.2542	−0.3592	0.1404	0.042*	
H12B	−0.3134	−0.3436	0.2416	0.042*	
C13	−0.1623 (3)	−0.3018 (2)	0.2559 (7)	0.0398 (14)	
H13A	−0.1497	−0.3431	0.2227	0.048*	
H13B	−0.1349	−0.2919	0.3386	0.048*	
C14	−0.1470 (3)	−0.2560 (3)	0.1394 (6)	0.0389 (13)	
H14A	−0.1120	−0.2287	0.1734	0.047*	
H14B	−0.1298	−0.2790	0.0574	0.047*	
C15	−0.2500	−0.2500	0.0114 (7)	0.0314 (16)	
H15A	−0.2282	−0.2801	−0.0507	0.038*	0.5
H15B	−0.2718	−0.2199	−0.0507	0.038*	0.5

### Atomic displacement parameters (Å^2^)

**Table:**

	*U*^11^	*U*^22^	*U*^33^	*U*^12^	*U*^13^	*U*^23^
Br1	0.0464 (3)	0.0333 (2)	0.0323 (2)	0.0043 (3)	−0.0102 (3)	0.0045 (2)
O1	0.039 (3)	0.0219 (18)	0.0323 (19)	−0.0044 (18)	−0.0097 (17)	0.0049 (15)
C1	0.021 (3)	0.022 (2)	0.027 (2)	0.004 (2)	0.001 (2)	−0.0017 (19)
C2	0.032 (3)	0.020 (2)	0.034 (3)	−0.003 (2)	0.003 (2)	0.001 (2)
C3	0.034 (3)	0.020 (2)	0.030 (3)	−0.001 (2)	0.005 (2)	0.005 (2)
C4	0.029 (3)	0.024 (2)	0.021 (2)	0.004 (2)	0.004 (2)	0.0013 (18)
C5	0.026 (3)	0.0200 (18)	0.030 (2)	−0.0030 (18)	0.001 (3)	−0.002 (3)
C6	0.033 (3)	0.018 (2)	0.027 (2)	−0.003 (2)	0.002 (2)	0.0007 (18)
N11	0.032 (2)	0.0210 (18)	0.019 (2)	−0.0012 (17)	0.0013 (17)	−0.0048 (15)
N12	0.025 (2)	0.036 (2)	0.0222 (19)	−0.009 (2)	−0.0016 (18)	−0.0016 (17)
C11	0.043 (5)	0.024 (3)	0.016 (3)	0.005 (3)	0.000	0.000
C12	0.050 (4)	0.027 (3)	0.029 (3)	−0.014 (3)	−0.006 (2)	−0.001 (2)
C13	0.034 (3)	0.041 (3)	0.045 (4)	0.006 (2)	0.002 (3)	0.004 (3)
C14	0.027 (3)	0.061 (4)	0.029 (3)	−0.006 (3)	0.003 (2)	0.001 (3)
C15	0.034 (4)	0.046 (4)	0.015 (3)	−0.006 (3)	0.000	0.000

### Geometric parameters (Å, º)

**Table:**

Br1—C4	1.907 (5)	N12—C15	1.453 (6)
O1—C1	1.355 (6)	N12—C14^i^	1.462 (8)
O1—H1	0.78 (7)	C11—N11^i^	1.456 (5)
C1—C2	1.388 (7)	C11—H11A	0.9900
C1—C6	1.393 (7)	C11—H11B	0.9900
C2—C3	1.380 (8)	C12—H12A	0.9900
C2—H2	0.9500	C12—H12B	0.9900
C3—C4	1.381 (7)	C13—C14	1.520 (8)
C3—H3	0.9500	C13—H13A	0.9900
C4—C5	1.384 (7)	C13—H13B	0.9900
C5—C6	1.380 (8)	C14—N12^i^	1.462 (8)
C5—H5	0.9500	C14—H14A	0.9900
C6—H6	0.9500	C14—H14B	0.9900
N11—C11	1.456 (5)	C15—N12^i^	1.453 (6)
N11—C13	1.470 (8)	C15—H15A	0.9900
N11—C12	1.482 (7)	C15—H15B	0.9900
N12—C12	1.441 (7)		
			
C1—O1—H1	107 (5)	N11—C11—H11B	107.5
O1—C1—C2	117.4 (5)	N11^i^—C11—H11B	107.5
O1—C1—C6	123.1 (5)	H11A—C11—H11B	107.0
C2—C1—C6	119.5 (5)	N12—C12—N11	119.5 (4)
C3—C2—C1	120.4 (5)	N12—C12—H12A	107.4
C3—C2—H2	119.8	N11—C12—H12A	107.4
C1—C2—H2	119.8	N12—C12—H12B	107.4
C2—C3—C4	119.6 (4)	N11—C12—H12B	107.4
C2—C3—H3	120.2	H12A—C12—H12B	107.0
C4—C3—H3	120.2	N11—C13—C14	116.4 (5)
C3—C4—C5	120.8 (5)	N11—C13—H13A	108.2
C3—C4—Br1	119.8 (4)	C14—C13—H13A	108.2
C5—C4—Br1	119.4 (4)	N11—C13—H13B	108.2
C6—C5—C4	119.6 (4)	C14—C13—H13B	108.2
C6—C5—H5	120.2	H13A—C13—H13B	107.3
C4—C5—H5	120.2	N12^i^—C14—C13	116.6 (5)
C5—C6—C1	120.1 (4)	N12^i^—C14—H14A	108.1
C5—C6—H6	119.9	C13—C14—H14A	108.1
C1—C6—H6	119.9	N12^i^—C14—H14B	108.1
C11—N11—C13	113.2 (3)	C13—C14—H14B	108.1
C11—N11—C12	115.3 (4)	H14A—C14—H14B	107.3
C13—N11—C12	113.9 (4)	N12^i^—C15—N12	118.2 (6)
C12—N12—C15	114.7 (4)	N12^i^—C15—H15A	107.8
C12—N12—C14^i^	114.9 (4)	N12—C15—H15A	107.8
C15—N12—C14^i^	114.8 (4)	N12^i^—C15—H15B	107.8
N11—C11—N11^i^	119.3 (5)	N12—C15—H15B	107.8
N11—C11—H11A	107.5	H15A—C15—H15B	107.1
N11^i^—C11—H11A	107.5		
			
O1—C1—C2—C3	177.5 (5)	C12—N11—C11—N11^i^	−51.2 (3)
C6—C1—C2—C3	−2.7 (8)	C15—N12—C12—N11	55.6 (7)
C1—C2—C3—C4	1.0 (8)	C14^i^—N12—C12—N11	−80.7 (7)
C2—C3—C4—C5	0.7 (8)	C11—N11—C12—N12	50.6 (7)
C2—C3—C4—Br1	−178.0 (4)	C13—N11—C12—N12	−82.8 (6)
C3—C4—C5—C6	−0.6 (8)	C11—N11—C13—C14	−69.9 (6)
Br1—C4—C5—C6	178.1 (4)	C12—N11—C13—C14	64.4 (6)
C4—C5—C6—C1	−1.2 (8)	N11—C13—C14—N12^i^	2.4 (7)
O1—C1—C6—C5	−177.4 (5)	C12—N12—C15—N12^i^	−53.9 (3)
C2—C1—C6—C5	2.8 (8)	C14^i^—N12—C15—N12^i^	82.4 (4)
C13—N11—C11—N11^i^	82.4 (4)		

Symmetry code: (i) −*x*−1/2, −*y*−1/2, *z*.

### Hydrogen-bond geometry (Å, º)

**Table:**

*D*—H···*A*	*D*—H	H···*A*	*D*···*A*	*D*—H···*A*
O1—H1···N11	0.78 (7)	1.97 (7)	2.705 (5)	158 (7)
C3—H3···O1^ii^	0.95	2.42	3.347 (6)	164
C13—H13*A*···Br1^iii^	0.99	2.89	3.833 (6)	159

Symmetry codes: (ii) −*x*−1/2, −*y*−1, *z*+1/2; (iii) *x*, *y*, *z*−1.
